# Intention to Use Maternal Waiting Homes and Related Factors among Pregnant Women in Metu Woreda, Western Ethiopia

**DOI:** 10.4314/ejhs.v32i5.2

**Published:** 2022-09

**Authors:** Worke Yismaw, Tigist Teklu, Addishiwot Fantahun, Boka Dugassa, Rodas Merid, Ketema Bizuwork

**Affiliations:** 1 Metu University College of Health Sciences, Metu, Ethiopia; 2 Addis Ababa University College of Health Sciences, Addis Ababa, Ethiopia; 3 St. Paul Millennium Medical College, Addis Ababa, Ethiopia

**Keywords:** Maternal Waiting Homes, Intention to Use, Pregnant Women, Ethiopia

## Abstract

**Background:**

Maternity Waiting Homes are houses built in the healthcare settings that lodge pregnant women in their term state of pregnancy to prevent labor and delivery-related complication. This study aimed to estimate the extent of pregnant women's intention to use Maternal Waiting Homes and identify its associated factors in Metu Woreda, Western Ethiopia.

**Methods:**

A community-based cross-sectional study was conducted from March 1–30, 2018. We used a systematic sampling method to select the study participants and Binary logistic regression analysis was used to identify factors associated with the intention of the women to use Maternal Waiting Homes.

**Results:**

A total of (97%) of respondents' questionnaires were found complete and analyzed for this study. Almost half (48.8%) of the pregnant women who participated in the study were planned to use Maternal Waiting Homes in their prospective delivery. Based on multivariate logistic regression analysis; being illiterate and/or less educated in their educational status, having a history of using Maternal Waiting homes, and receiving a number of times antenatal care services were found statistically significantly associated with intention of the women to use Maternal Waiting Homes.

**Conclusions:**

It is trivial that more than half of the pregnant women who participated in the study were unintended to use Maternal Waiting Homes. Educational status, a number of times attending antenatal care services and experience of using Maternal Waiting Homes were found statistically significantly associated with women's intention to use Maternal Waiting Homes.

## Introduction

Incontrovertibly, maternal mortality reduction is a global priority (1). However, global maternal mortality is still high in developing countries (2,3). According to the World Health Organization report of 2017, an estimated 295, 000 maternal deaths were recorded with almost 94% of them were from poor-income countries (4). For example, sub-Saharan African and South Asian countries account for over 85%, with almost three fourth of them were from Sub-Saharan African countries, including Ethiopia (2,4). Ethiopia is among ten countries that accounted for 60% of global maternal mortality in 2017 (2,4). According to the 2016 Ethiopian Demographic Health Survey report, Ethiopia has a high maternal mortality; an estimated at 412 per 100,000 live births (5). This high prevalence of maternal mortality is due to less use of institutional delivery (6) and/or delays in the utilization of emergency obstetric care (7) which results in high maternal mortality (2,8,9) through inappropriate management of labor and delivery-related complications like postpartum hemorrhage, eclampsia/preeclampsia, infections like sepsis, and unsafe abortion (10,11). World Health Organization recommended Maternal Waiting Homes (MWHs) (2,8,9,12) for women to easily access professional healthcare that would be delivered by healthcare institutions.

MWHs are residential houses where women who live remotely can wait before giving birth at health facilities (9). MWHs relieve problems women encounter to get healthcare access given by healthcare facilities, which may reduce labor and delivery-related complications (8,13,14). Principally, this strategy (Use of Maternal Waiting Homes) is developed for women living in remote areas where transportation is inaccessible, difficult, and/or very slow (12,15). MWHs are essential to decrease maternal and neonatal mortality because it avoids delays in accessing obstetric care delivered by healthcare professionals in the healthcare institutions (12). Pregnant women who used MWHs can easily access essential childbirth care, obstetric, and/or newborn care services at the nearby healthcare facility (16).

The use of Maternal Waiting Homes in the healthcare institution and/or nearby the healthcare institution is among the existing strategies recommended by the World Health Organization to reduce maternal and perinatal mortality (15,17), especially in the low and middle-income countries (18). Previously, this program/strategy targeted pregnant women with the risk of maternal complications (19–21), however, currently, it addresses all pregnant women (22,23). The strategy was implemented in Africa as the “Campaign on Accelerated Reduction of Maternal, Newborn & Child Mortality in Africa (CARMMA)” launched in May 2009, during the African Union 4^th^ Conference of Ministers of Health (CAMH4) held in Addis Ababa, Ethiopia with the campaign's slogan: ‘Africa Cares: No Woman Should Die While Giving Life’ (24).

Ethiopia started executing Maternal Waiting Homes in 1976 (9) via the use of usual chalets in the local area to connect the physical distance between the healthcare facilities and communities. The government of Ethiopia launched the national expansion program regarding MWHs at the country level in 2014 (25). The current Ethiopian health strategic plan was planned to launch Maternity Waiting Homes in 75% of healthcare centers by 2020 (26), conversely, several challenges like lack of transportation for mothers to their homes, lack of healthcare givers at the healthcare centers, inadequacy and sub-optimal quality of food provided by Maternity Waiting Homes, inappropriate targeting of women in need of the service, low-quality Maternal Waiting Homes built (26) distance from a residential home, high cost and long time to access the healthcare (14), women's negative attitude towards Maternal Waiting Homes(18) are among the most common factors that hinders women not to use Maternal Waiting Homes.

Despite studies conducted revealing the use of Maternal Waiting Homes has a significant impact on reducing perinatal and maternal mortality (12,27–29), some studies conducted in Ethiopia revealed only 42.6% to 65.3% (28–30) pregnant women have intention to use Maternal Waiting Homes in their prospective delivery (31) while one study from the western part of Ethiopia revealed 38.7% of the study participants had experience in using Maternal Waiting Homes. Evidencing benefits of the use of Maternal Waiting Homes (12,27–29) to avert labor and delivery-related complications that may occur during labor and delivery and/or postpartum period is well established elsewhere (32–34)

Further, the finding of this study provides information to the national/local policymakers, researchers, governments, and stakeholders, all of who deal to reduce maternal and/or neonatal mortality that may occur due to labor and delivery-related complications. However, little was known about the intention of pregnant women's use of Maternal Waiting Homes with their associated factors in the Western Ethiopia. The purpose of this study was to assess intention to Use Maternal Waiting Homes and its associated factors among pregnant women in Metu Woreda, Western Ethiopia. Note that, the current study used the theory of reasoned action and the health belief model as a base model (35,36).

## Methods and Materials

**Study Setting and Period**: The study was conducted in Metu Woreda; one of the Woreda in the Illu-Ababora zone of the Oromia Region, Ethiopia. According to the Ethiopian Central Statistical Agency report in 2019, the Woreda's population were 81994, among them 40, 842 were Males while 41, 152 were Females (37). The Woreda has twenty-eight administrative Kebeles (Smallest Administrative Units in the Country). According to the 2017 first quarter health office report of the Woreda; there were about 2067 pregnant women in the Woreda. The study was conducted from March 01-30/2018.

**Study Design, Study Participants, and Recruitment Procedure**: The community-based cross-sectional study design was used to recruit the study participants. The source populations were all pregnant women living in Mettu Woreda while the study participants were pregnant women excluding those who were critically sick, unable to respond to the study questionnaire, and don't want to participate in the study. The sample size was calculated using a single population proportion and correction formula by considering the previous extent of intention of pregnant women to use Maternal Waiting Homes (57.3%) (29), 95% confidence level to show the estimated falls between the upper and lower limits specified by the confidence interval with a marginal error of 5%, 1.5 design effect to adjust the calculated sample size for clusters of pregnant women formation, and 5% non-response rate. Therefore, the final calculated sample size was 501(Five hundred one). Then, the calculated sample size was proportionally allocated to the Kebeles (Smallest Administrative Units of the Country) based on the total number of pregnant women living in the Kebeles. After the total number of pregnant women living in the Woreda was divided by the calculated sample size to get the K (constant) value, which was four, a systematic sampling method was used to select the study participants to the study. To make it briefer, first, a cluster of pregnant women formed based on their similar characteristics were selected as study units by a simple random sampling method. Then, the first study participant was selected using the lottery method from one of the study units and continued to select the pregnant women to include in the study every four pregnant women from the listed name of the pregnant women in the study units.

**Data Collection Tool, Quality Control, and Data Collection Procedures**: The interview guide consists of socio-demographic, obstetric history, and intention to use Maternal Waiting Homes. Data was collected through face-to-face interviews with a pre-tested interview guideadapted from a study conducted in Jimma town (7). The data collection tool was translated into the local language Afaan Oromo and retranslated to the English language by experts on the subject matter. Additionally, the study tool was checked for reliability by a Cronbach alpha score and rated at 0.81. The validity of the data collection tool was checked by the subject matter experts, and necessary modifications were made based on the suggestion and comments of the experts. Seven data collectors and two supervisors who speak the local language Afaan Oromo comprised of Midwifery and Nurses were deployed for data collection after two days of extensive training.

**Outcome Variable and its Measurement Methods:**

**Intention to Use Maternal Waiting Homes**: Intention of pregnant women to use Maternal Waiting Homes in their prospective delivery was measured using the following questions: 1) I intended to use Maternal Waiting Homes two to four weeks before the expected date of delivery of my current pregnancy, 2) I will make my effort to use Maternal Waiting Homes for my current pregnancy, 3) I plan to use Maternal Waiting Homes for my current pregnancy, 4) I like to use Maternal Waiting Homes for fifteen days in my current pregnancy. The questions were arranged in five Likert scale questions (1: Strongly disagree, 2: Disagree, 3: Neutral, 4: Agree, 5: Strongly agree). Total score ranges from four to twenty scores. A total score greater than or equal to the mean score was revealed as intended to use Maternal Waiting Homes. The cutoff point declares whether pregnant women were intended to use Maternal Waiting Homes or not is summarized in table **1** below.

**Table 1:** Cut-off point Score for Intention to use Maternal Waiting Homes

Variable	Cut off point Score	Level
**Intention to use MWH**	≥ 12	Intended to use MWH
	< 12	Unintended to use MWH

Note: MWH = Maternal Waiting Homes

**Definition of Variables**

- Intention to Use Maternal Waiting Home is defined as pregnant women's willingness, and effort of planning to be usedand exert to utilize it (28).
- Maternity Waiting Homes are defined as temporary shelters in the healthcare settings or nearby the healthcare settings where women stay before and occasionally after given a birth (15).
- Socio-demographic is defined as characteristics of the population like age, gender, ethnicity, educational level, income, type of client, years of experience, etc. (38).
  Obstetric History is defined as relevant information related to a patient's current and previous pregnancies (39).

**Data Processing and Analysis:** The collected data were entered twice, cleaned, and edited using Epi data version 3.1 statistical software and analyzed with SPSS version 21. Descriptive statistics were used to describe the variables while binary logistic regression analysis was used to check the association of variables with intention of the women to use Maternal Waiting Homes. The significance level was declared at P-value < 0.05. To examine the factors associated with intention of the women to use Maternal Waiting Homes, we used multivariate logistic regression analysis.

**Ethical Considerations**: The ethical approval letter was obtained from the College of Health Sciences, Addis Ababa University, and an official letter of cooperation was written to the Metu Woreda Health Office from the University. After receiving a letter of cooperation and the ethical approval letter from the University, Metu Health Office wrote a letter of support based on the reference letter written from the University. Verbal consent and assent were obtained from each study participant. Also, affirmation was given to discontinue participating in the study without any form of prejudice made.

## Results

**Background characteristics of the respondents**: Among the total of pregnant woman participated in the study, 97% were completed the interview session. The majority (45.7%) were younger (15–24). The mean age was 25.3±5.7. More than half (58.8%) of the participants were attended primary education. Moreover, over three fourth (91.6%) of the study participants were found married (Table 2).

**Table 2:** Background characterstics (N=490)

Variable	Frequency
Age in Years	
15–24	224 (45.7)
25–34	216 (44.1)
≥ 35 Table	50 (10.2)
Mean age and SD	25.3±5.7
Religion	
Orthodox	362 (73.9)
Muslim	128 (26.1)
Educational Status	
Unable to Read and Write	98 (20)
Primary Education	288 (58.8)
Secondary Education & Above	104 (21.2)
Marital Status	
Married	449 (91.6)
Unmarried	41 (8.4)
Current Occupation	
Unemployed	467 (95.3)
Employed	23 (4.7)
Monthly Income	
< 1000 EB	316 (64.5)
≥ 1000 EB	174 (35.5)
History of Gravidity	
≤ 2	242 (49.4)
≥ 3	248 (50.6)
History of Antenatal Visit	
Didn't Attend any ANC Visit	76 (15.5)
≤ 2nd visit	168 (34.3)
≥ 3rd visit	246 (50.2)
History of Parity	
Yes	350 (71.4)
No	140 (28.6)
Past Experience of Maternal	
Waiting Homes	
Yes	146 (29.8)
No	344 (70.2)
Experience in Institutional Delivery	
Yes	186 (38)
No	304 (62)

Note: Abbreviations, EB = Ethiopian Birr; N = Number; Marital Status: Unmarried Includes Single, Widowed, Divorced, Occupation: Unemployed Includes House Wife, Farmers and Students, ANC = Antenatal Care. MWH = Maternal Waiting Homes, SD = Standard Deviation

**Intention to Use Maternity Waiting Homes**: This study found almost half (48.8%) of the pregnant women who participated in the study were intended to use Maternal Waiting Homes. The majority of pregnant women who intended to use Maternal Waiting Homes were were from age group 15–24 years. The majority of pregnant women who intended to use Maternal Waiting Homes were attended primary educational level. Further, among pregnant women who intended to use Maternal Waiting Homes, a significant number of them had an experience of using Maternal Waiting Homes in their earlier labor and delivery period (Table 3).

**Table 3:** Association of Factors under Bivariate and Multivariate Logistic Regression Analysis with an Intention to Use Maternal Waiting Homes (N=490)

Variables	Intention to use Maternal Waiting Homes			
**Variable**	Yes(239)	No(251)	COR(95%CI)	AOR(95%CI)
	No. (%)	No. (%)		
**Age**				
**15–24yrs**	107	117	1.6[0.88, 3.10]	
**25–34yrs**	102	114	1.70[0.90, 3.13]	
**≥ 35yrs**	30	20	1	
**Marital status**				
**Married**	222	227	0.72[0.38, 1.39]	
**Unmarried**	17	24	1	
**Educational status**				
**Unable to read and** **write**	36	62	2.17[1.24, 3.82]*	2.13[1.18, 3.85]*
**Primary education**	145	143	1.24[0.79, 1.95]	
**≥ Secondary education**	58	46	1	
**Occupation**				
**Unemployed**	229	238	0.80[0.34, 1.86]	
**Employed**	10	13	1	
**Experience in using**				
**Yes**	104	42	0.26[0.17, 0.40]*	0.27[0.18, 0.42]*
**No**	135	209	1	
**History of antenatal visit**				
**Didn't attend any ANC** **visit**	22	54	0.34(0.21, 0.62)*	2.26[1.26, 4.06]*
**≤ 2nd visit**	86	82	0.92(0.62, 1.36)	
**≥ 3rd visit**	131	115	1	
**History of parity**				
**Yes**	186	164	1.86(1.25, 2.78)*	
**No**	53	87	1	1

* Statistically Significantly Associated at P < 0.05

1 = Reference, MWH = Maternal Waiting Homes, ANC = Antenatal Care

**Association of Variables with Intention to use Maternal Waiting Homes**: To measure the association of variables with intention of the pregnant women to use Maternal Waiting Homes, we used binary logistic regression analysis. Under multivariate logistic regression analysis, we found the not/or less educated pregnant women [AOR = 2.13(95% CI: 1.18, 3.85), P <0.03] were found more likely intended to use Maternal Waiting Homes compared to pregnant women more educated. Nevertheless, having experience of using Maternal Waiting Homes in their earlier labor and delivery period [AOR = 0.27(95% CI: 0.18, 0.42), P< 0.01] were found less likely intended to use Maternal Waiting Homes. Moreover, pregnant women who used a greater number of antenatal care service visits [AOR = 2.26(95% CI: 1.26, 4.06), P< 0.01] were found more likely intended to use Maternal Waiting Homes (Table 3). Further, the variance of the three statistically significantly associated variables with the dependent variable intention of pregnant women to use Maternal Waiting Homes were checked for the model fit test and rated at R^2^ = 0.11; which shows variables statistics were fit for regression analysis.

## Discussion

Access to healthcare facilities through the use of Maternal Waiting Homes; indisputably reduces obstetric complications related to pregnancy, labor, and/or delivery (40). Therefore, it is important to measure the intention of pregnant women to use Maternal Waiting Homes for their prospective delivery.

This study found almost half (48.8%) (95% CI: 0.43 to 0.55) of the pregnant women were intended to use Maternal Waiting Homes for their prospective delivery. This indicates over half of the pregnant women participated in the study were unintended to use Maternal Waiting Homes for their prospective delivery, which may result in a significant rate of maternal and/or neonatal morbidity and mortality. Therefore, it is worrisome that more than half of the pregnant women who participated in the study were unintended to use Maternal Waiting Homes despite high neonatal and maternal morbidity and mortality in the Region (26,41).

The primary reasons makes women not intend to use maternal waiting homes would be low awareness of the community about the use of Maternal Waiting Homes, low/substandard Maternal Waiting Homes built in the healthcare institution, myths about Maternal Waiting Homes in the community, lack of awareness about the existence of Maternal Waiting Homes in the healthcare settings or nearby the healthcare settings, the women may fail to decide by themselves to use Maternal Waiting Homes, long distance to reach the health facility. Some of these reasons were cited as reasons hindering the women not to use Maternal Waiting Homes elsewhere (15,42). This finding is in line with a study from the Gamo-Gofa zone, Southern part of Ethiopia (28). This may be due to the healthcare professionals in the two study settings were motivating the women to use Maternal Waiting Homes at equivalent levels, may the communities living in the two study settings have similar socio-demographic characteristics, and/or quality of Maternal Waiting Homes built was found in the same level. This finding is lower than a study conducted in Jimma Woreda southwest of Ethiopia (7) and a study conducted in Butajira, Ethiopia (25). However, it is higher than a study conducted in rural health centers of Ethiopia (43), a study conducted in Bench-Majizone, Southwest Ethiopia revealed (39%) of women who participated in the study had used Maternal Waiting Homes in their previous delivery (30), a study conducted in Jimma zone revealed only 7% of the women participated in the study had the experience of using Maternal Waiting homes (44), a review paper from Ethiopia (45) a study from Kenya (42), Zambia (46). This difference could be due to differences in the sample size, study period, availability of health extension workers in the respective Woreda, and Maternal Waiting Homes in the healthcare settings and/or nearby the healthcare settings of communities of this study. The reasons for the difference in the intention of pregnant women to use Maternal Waiting Homes were partly described by other studies of developing countries (30,42).

This study revealed factors associated with the intention of the pregnant women to use Maternal Waiting Homes were educational status, experience of using Maternal Waiting Homes, and several Antenatal Care Service Visits. Partly, the same findings were reported from previous three studies from Ethiopia and one study from Zambia (25,29,40,44).

Unlike previous studies (25,29,44), this study revealed that less-educated pregnant women who participated in the study were more likely intended to use Maternal Waiting Homes compared to the more educated ones. This may be due to strong home-to-home follow-up of health extension workers of the Woreda. Moreover, regarding the experience of using Maternal Waiting Homes, this study revealed pregnant women who used Maternal Waiting homes in their prior delivery period were less likely to use Maternal Waiting homes for their current delivery period. This may be due to the Maternal Waiting Homes built in the region is low/substandard, less satisfaction level of the comprehensive care given in the Maternal Waiting Homes, fear of exhaustion faced to the far distance from their residence area, preferring food served at home. This finding is in line with a previous study from Ethiopia (7). To the contrary of the finding of this study, a study from Zambia revealed having a history of using Maternal Waiting Homes was found to be more likely to use health facility services like antenatal, postnatal, and vaccination (40). Furthermore, this study found an increased number of attending antenatal care service results in more odds to intend to use Maternal Waiting Homes. This may be due to, the pregnant women getting enough information about birth preparedness, labor, and delivery-related complications through their increased number of antenatal care service visits, and may becoming more aware of the importance of Maternal Waiting Homes. This finding is in line with a study from the southeastern part of Ethiopia (30). This could be due to healthcare workers in both settings providing enough information about safe obstetric care, complications, and the purpose of Maternal Waiting Homes.

Therefore, bearing in mind the higher maternal mortality rate in developing and poor income countries including Ethiopia, and the low uptake of Maternal Waiting Homes by the pregnant women's the following recommendations can be made. First, the government, and/or stakeholders should continue to address the Maternal Waiting Homes at an approximate nearest distance to the residence of the communities and healthcare facilities. Second, the government and stakeholders should increase the way the community gets sufficient information about the benefit of Maternal Waiting Homes and possible labor and delivery-related complications that may occure due to delay in seeking obstetric care given in the healthcare institution and/or from taking the service from unskilled/unprofessional personnel. Third, the government and/or stakeholders should build standard/quality Maternal Waiting Homes, as this increases the intention of the pregnant women to use the Maternal Waiting Homes. Finally, the community administrators, and healthcare professionals should encourage and support the women to use Maternal Waiting Homes.

In conclusion, the study revealed over fifty percent of the pregnant women who participated in the study were unintended to use Maternal Waiting Homes in their prospective delivery. Further, having a history of using Maternal Waiting Homes, illiteracy and/or lesser educational status, and attending several antenatal care service visits were found factors affecting the intention of the pregnant women to use maternal waiting homes. Therefore, there should be an intervention to enhance the intention of the women to use the Maternal Waiting Homes, by considering the factors associated with the intention of the pregnant women to use Maternal Waiting Homes.
